# Dietary Patterns and Sociodemographic Determinants of Underweight and Excess Body Weight Among Nursing Students in Southern Thailand

**DOI:** 10.3390/ijerph23070900

**Published:** 2026-07-13

**Authors:** Naiyana Noonil, Hatwani Darakai, Muhammad Kamil Bin Che Hasan, Udomsak Narkkul, Pikuntip Kunset

**Affiliations:** 1 The Excellent Center of Community Health Promotion, Department of Community Nursing, School of Nursing, Walailak University, Nakhon Si Thammarat 80160, Thailand; nnaiyana@mail.wu.ac.th; 2 Praboromarajchanok Institute, Boromarajonani College of Nursing, Nakhon Si Thammarat 80000, Thailand; hatwani@bcnnakhon.ac.th; 3 Kulliyyah of Nursing, International Islamic University Malaysia, Kuantan 25200, Malaysia; mkamil@iium.edu.my; 4 Department of Medical Science, School of Medicine, Walailak University, Nakhon Si Thammarat 80160, Thailand; udomsak.na@wu.ac.th

**Keywords:** body mass index, dietary patterns, excess body weight, nursing students, Southern Thailand, underweight

## Abstract

**Highlights:**

**Public health relevance—How does this work relate to a public health issue?**

- Dietary patterns and lifestyle behaviors among future healthcare professionals may influence long-term health outcomes and health promotion practices.
- At the population level, underweight and excess body weight coexist among nursing students in Southern Thailand.
- Dietary patterns and lifestyle-related behaviors can inform student health and campus food environment initiatives.

**Public health significance—Why is this work of significance to public health?**

- Sedentary behavior, meal frequency, alcohol use, and academic factors were associated with dietary patterns among underweight and excess body weight students.
- Three empirically derived dietary patterns differed in food composition and showed distinct associations with sociodemographic and behavioral factors within underweight and excess-body-weight subgroups.
- The findings identify potentially modifiable targets for nutrition promotion among future health professionals.

**Public health implications—What are the key implications or messages for practitioners, policy makers and/or researchers in public health?**

- Nursing schools should integrate nutrition screening, practical meal planning support, and healthier campus food options into student wellness programs.
- Future multicenter longitudinal studies should examine dietary patterns alongside stress, body image, sleep, and disordered eating symptoms.

**Abstract:**

(1) Background: The double burden of malnutrition (DBM) occurs at the population level when undernutrition and overweight or obesity coexist. In this study, DBM was operationalized as BMI-defined underweight and excess body weight (EBW) within the sampled student population. (2) Objective: To identify dietary patterns and examine their associations with sociodemographic and health behavior factors among underweight and EBW nursing students in Southern Thailand. (3) Methods: This secondary analysis used cross-sectional data collected from 372 undergraduate nursing students in 2023. Dietary intake was assessed using a food frequency questionnaire; principal component analysis identified dietary patterns, and separate multivariable linear regression models examined associations with pattern scores in the underweight and EBW subgroups. (4) Results: Underweight and EBW were present in 22.0% and 26.3% of students, respectively. The snack pattern comprised milk and milk products, sweetened beverages, bakery items, desserts, pickled fruits, and snacks; the Thai dish pattern comprised rice, spicy curry, meat, entree-over-rice dishes, and vegetables; and the high-energy pattern comprised sticky rice and fried meat. Among underweight students, Thai dish scores were positively associated with year of study (*p* < 0.001), breakfast intake (*p* < 0.001), and supper intake (*p* = 0.008), and negatively associated with alcohol drinking (*p* = 0.014) and sedentary behavior (*p* = 0.018). Among students with EBW, snack pattern scores were higher among female students (*p* = 0.032) and students reporting exercise (*p* = 0.001) or sedentary behavior (*p* < 0.001), whereas Thai dish scores were associated with income (*p* = 0.006), dinner intake (*p* = 0.005), sex (*p* = 0.001), and supper intake (*p* = 0.034). Higher GPA (grade point average) was associated with lower high-energy-pattern scores in both subgroups (underweight, *p* = 0.018; EBW, *p* = 0.007). (5) Conclusion: BMI-defined underweight and EBW coexisted in this population. Dietary pattern correlates differed by nutritional status subgroup, underscoring the need for tailored nutrition and lifestyle interventions, as well as healthier university food environments.

## 1. Introduction

The double burden of malnutrition (DBM) is an important public health challenge during the nutrition transition in low- and middle-income settings. The World Health Organization describes DBM as the coexistence of different forms of malnutrition, including undernutrition and overweight or obesity, within individuals, households, or populations [1,2]. In the present study, DBM is used at the population level: it denotes the simultaneous presence of BMI-defined underweight and excess body weight (EBW) among the sampled nursing students, rather than the presence of both conditions in the same individual. BMI was classified using Asian cut-offs [3]. University students may be vulnerable to both forms of malnutrition. Across university student samples, underweight prevalence has ranged from 6.9% to 15.2%, whereas overweight and obesity prevalence have ranged from 10.4% to 24.5% [4,5,6,7,8]. The transition to university may alter meal patterns, physical activity, food purchasing, and sleep-related routines [9,10,11,12].

Nursing students are future healthcare professionals who are expected to model health-promoting behaviors, including healthy eating practices [13]. Nevertheless, dietary intake in this group may be shaped by time pressure, clinical placements, social relationships, financial resources, and food availability [14,15]. Describing dietary patterns, rather than isolated foods or nutrients, can therefore provide a more meaningful picture of habitual dietary behavior and its social context.

The transition to university may be accompanied by stress, financial pressure, and social adjustment. These factors have been associated in previous studies with irregular meals, low physical activity, and greater reliance on energy-dense convenience foods [9,10,11,12]. Such pathways were not directly measured in the present study and are considered possible explanations rather than study findings.

Previous research among university and health science students has identified diverse dietary patterns, ranging from more traditional or plant-rich patterns to patterns characterized by sugar-sweetened beverages, processed foods, bakery products, or other energy-dense foods [5,6,7,8,14]. Sociodemographic characteristics and lifestyle behaviors have also been associated with dietary patterns and nutritional status [5,6,7,8,14,16]. Inadequate intake of protein and micronutrient-rich foods may compromise nutritional status [17,18]. Dietary habits have also been associated with academic performance and weight-related outcomes among university students [5,14,19]. Nursing students may encounter additional challenges during clinical training and in demanding healthcare environments, which can make it harder to maintain healthy lifestyle behaviors [20]. However, few studies have examined dietary pattern correlates separately among students with underweight and those with EBW, particularly among nursing students in Thailand.

Southern Thailand provides a relevant setting because Buddhist and Muslim communities coexist, and local food practices may vary across campuses and communities. The present study does not measure cultural practices or dietary restrictions; therefore, any observed association with religion should be interpreted as a marker of unmeasured social and cultural context rather than evidence of a specific religious dietary practice.

We hypothesized that dietary pattern scores would be associated with sociodemographic characteristics and health behaviors among nursing students with underweight status and EBW. Accordingly, this study aimed to identify dietary patterns and examine their associations with sociodemographic and health behavior factors in these two nutritional status subgroups in Southern Thailand.

## 2. Materials and Methods

### 2.1. Study Design and Setting

This secondary analysis used cross-sectional survey data collected between July and August 2023 from undergraduate nursing students in Southern Thailand. The sampling frame comprised 2337 students enrolled in five nursing colleges located in upper and lower Southern Thailand.

A multistage sampling design was used. The five colleges were stratified into upper- and lower-southern regions, and one college was selected by simple random sampling from each stratum. Thus, two nursing colleges—one in Nakhon Si Thammarat Province and one in Songkhla Province—were included in the primary survey.

### 2.2. Participants and Eligibility Criteria

Eligible participants were first- to fourth-year undergraduate nursing students enrolled during the first semester of the 2023 academic year. At the primary data collection stage, participants provided written informed consent. The present study analyzed de-identified secondary data from 396 students who completed the questionnaire.

The primary survey excluded students with an acute illness or injury at the time of data collection, a chronic condition likely to affect dietary intake or nutritional status, a specific dietary restriction, or medication use likely to affect appetite or body weight. For the present analysis, records were excluded if the food frequency questionnaire (FFQ) was incomplete or if anthropometric data required to calculate BMI were missing.

### 2.3. Sample Size and Sampling Technique

The target population comprised 2337 nursing students from five colleges in Surat Thani, Nakhon Si Thammarat, Songkhla, Trang, and Yala provinces. The minimum sample size was calculated using the Krejcie and Morgan formula, assuming a population of 2337, a 95% confidence level (chi-square = 3.841), a population proportion of 0.50, and a margin of error of 0.05, yielding a minimum sample size of 330 students [21]. Allowing 20% for incomplete data or nonresponse, the target sample was 396 students.

**Figure 1:**
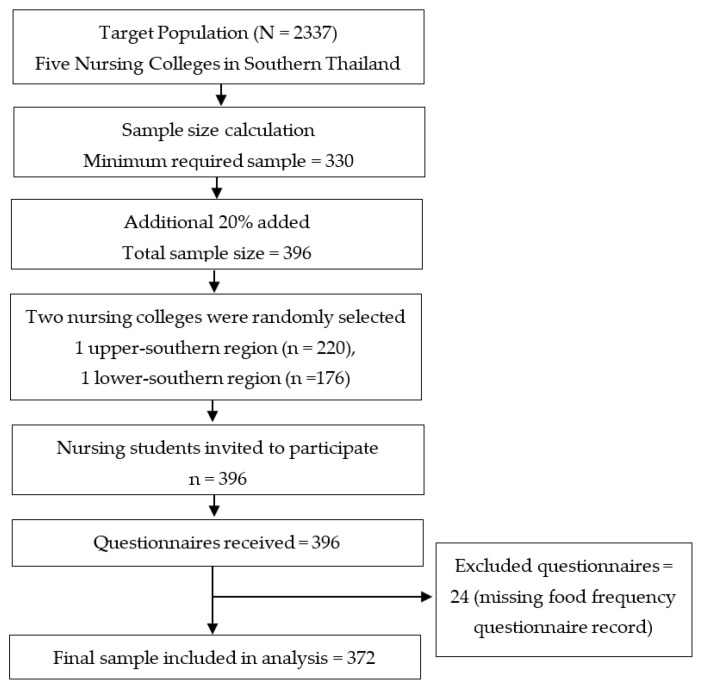
The flow diagram of the sample processes.

After two colleges were selected, the planned sample was allocated by college and year of study among the 1124 eligible students enrolled at those colleges. The planned allocation was 220 students from the upper-southern college and 176 from the lower-southern college; by year of study, the allocation was 106 first-year, 106 second-year, 78 third-year, and 106 fourth-year students. Students were selected from enrolment lists via simple random sampling within these strata. All 396 invited students returned questionnaires. Twenty-four records (6.1%) had incomplete FFQ information and were excluded because dietary pattern scores could not be derived, leaving 372 students (93.9% of returned questionnaires) for analysis (Figure 1).

### 2.4. Measures

#### 2.4.1. Sociodemographic and Health Behavior Data

Sociodemographic and health behavior data were collected using a self-administered questionnaire. Sociodemographic variables were sex, age, religion, year of study, grade point average (GPA; ≤3.00 or >3.00), and monthly personal income (≤5000 THB or >5000 THB, equivalent to approximately 155 USD at an exchange rate of 32 THB per dollar). The 5000 THB threshold was specified in the original questionnaire as a practical monthly allowance category and is consistent with allowance ranges reported among nursing students in the region [22]. Health behavior variables were tea or coffee drinking (yes/no), alcohol drinking (yes/no), exercise (yes/no), sedentary behavior (yes/no), and intake of breakfast, brunch, lunch, dinner, and supper. Meal frequency was categorized as ≤3 times per week or more than 3 times per week. Tea and coffee consumption was treated as a lifestyle-related dietary behavior because these beverages are commonly consumed by university students and may cluster with broader food and lifestyle practices [23]. All variables were analyzed as categorical variables.

#### 2.4.2. Anthropometric Measurements

Anthropometric measurements were taken by trained researchers using a standardized protocol. Weight was measured with a calibrated Tanita scale to the nearest 0.1 kg; the scale was checked by a laboratory officer. Students removed footwear, wore light clothing, stood upright with arms at their sides, and stepped off the scale before the value was recorded. Standing height was measured with a stadiometer to the nearest 0.1 cm while students removed footwear and headgear, stood erect with knees straight, and looked forward. BMI was calculated as weight in kilograms divided by height in meters squared. Based on Asian BMI criteria, participants were classified as underweight (<18.5 kg/m^2^), normal weight (18.5–22.9 kg/m^2^), or EBW (≥23.0 kg/m^2^), with EBW comprising overweight (23.0–24.9 kg/m^2^) and obesity (≥25.0 kg/m^2^) [3].

#### 2.4.3. Dietary Intake Assessment and Food Grouping

Dietary intake was assessed using a validated FFQ adapted from the Thai Food Consumption Survey [24]. Participants reported their usual food consumption during the preceding month; therefore, the frequency categories reflect habitual intake rather than consumption over a single week. The FFQ included 56 food items. Before PCA, individual foods were grouped into 20 mutually exclusive food groups based on food type, customary culinary use, and broad nutritional characteristics (Table 1) [25]. Intake frequency was scored as never (0), 1–3 times per week (1), 4–6 times per week (2), or daily (3). Food group scores were standardized to z-scores before PCA.

The FFQ had a content validity index of 1.00 and a Cronbach’s alpha coefficient of 0.80. Trained research assistants collected the data under the supervision of a nutrition expert.

### 2.5. Data Collection Procedure

At the primary data collection stage, questionnaires were completed in classrooms after written informed consent was obtained. Anthropometric measurements were undertaken on the same day. Research assistants were trained before data collection to promote consistent administration of the questionnaire and measurement procedures.

### 2.6. Statistical Analysis

Continuous variables were assessed for normality using the Kolmogorov–Smirnov test. Because age, GPA, monthly income, and BMI were non-normally distributed, they are reported as medians and interquartile ranges (IQRs). Categorical variables are presented as frequencies and percentages. Pearson’s chi-square test was used to compare the distribution of sociodemographic and health behavior variables across the underweight, normal weight, and EBW groups; Fisher’s exact test was used when expected cell counts were small. These were omnibus tests, and post hoc pairwise comparisons were not performed.

PCA with varimax rotation was used to derive dietary patterns from the 20 standardized food group scores [26]. Data suitability was assessed using the Kaiser–Meyer–Olkin (KMO) measure of sampling adequacy (0.558) and Bartlett’s test of sphericity (χ^2^ = 1696.487, *p* < 0.001). Components were retained when their eigenvalues exceeded 1.0, and the scree plot supported this decision. Factor loadings with an absolute value ≥0.30 were used to label and interpret patterns; the full loading matrix, including negative loadings, is presented in Table 2. Three components were retained: snack (eigenvalue 2.004), Thai dish (1.979), and high energy (1.861). Together, they explained 29.22% of the total variance. Regression-method factor scores were calculated for each participant.

Separate multivariable linear regression models examined associations between dietary pattern scores and selected sociodemographic or health behavior variables among underweight students and students with EBW. The normal weight group was included in descriptive and chi-square analyses but not in these planned subgroup models. Categorical predictors were coded as stated in the footnotes for Table 4 and Table 5; year of study was modeled as an ordinal variable (1–4). The results are reported as unstandardized regression coefficients (β), standard errors (SEs), and 95% confidence intervals (CIs). Given the subgroup sample sizes and number of predictors, these models were interpreted as exploratory. All tests were two-tailed, and *p* < 0.05 was considered statistically significant. Analyses were conducted using IBM SPSS Statistics, version 23 (IBM Corp., Armonk, NY, USA). 

### 2.7. Ethical Considerations

This study was a secondary analysis of de-identified data from a prior survey. The secondary data analysis was conducted in accordance with the Declaration of Helsinki and was approved by the Human Research Ethics Committee of Walailak University (protocol code WUEC-26-006-01; approved on 5 January 2026). At the primary data collection stage, students provided written informed consent; no additional consent was required for the de-identified secondary analysis.

## 3. Results

### 3.1. Characteristics of Participants

Of the 396 returned questionnaires, 24 had incomplete FFQ records and were excluded; 372 students were included in the analysis (Figure 1). The participants were 18–24 years old; the median age was 20 years (IQR 19–21), median GPA was 3.31 (IQR 3.08–3.57), median monthly income was 4000 THB (IQR 3000–5000), and median BMI was 20.41 kg/m^2^ (IQR 18.59–23.05). Students from all four academic years were represented: 99 (26.6%) first-year, 99 (26.6%) second-year, 75 (20.2%) third-year, and 99 (26.6%) fourth-year students. Most participants were female (92.7%).

Overall, 82 students (22.0%) were underweight, 192 (51.6%) had normal weight, and 98 (26.3%) had EBW; the latter group comprised 56 overweight students (15.1%) and 42 (11.3%) with obesity. Most participants had a GPA > 3.00 (80.4%) and a monthly income ≤ 5000 THB (80.4%).

The overall distribution of nutritional status differed by year of study (χ^2^ = 16.128, *p* = 0.013) and monthly income (χ^2^ = 11.202, *p* = 0.004). Descriptively, underweight was most frequent among first-year students (28.3%), whereas EBW was most frequent among second- and fourth-year students (34.3% in each group). EBW was also more frequent among students reporting a monthly income >5000 THB than among those reporting lower income (35.6% vs. 24.1%). Because no post hoc comparisons were performed, category-specific percentages should be interpreted descriptively.

Nutritional status distributions also differed by alcohol drinking (Fisher’s exact *p* = 0.034), reported exercise (χ^2^ = 7.529, *p* = 0.023), breakfast intake (χ^2^ = 10.080, *p* = 0.006), brunch intake (χ^2^ = 6.602, *p* = 0.037), and supper intake (χ^2^ = 6.089, *p* = 0.048). The complete distributions and omnibus test results are presented in Table 3.

### 3.2. Dietary Pattern

The data were suitable, although only marginally so, for factor analysis (KMO = 0.558; Bartlett’s test of sphericity χ^2^ = 1696.487, *p* < 0.001). PCA identified three dietary patterns that jointly explained 29.22% of total variance. The modest KMO value and variance explained indicate that these patterns should be interpreted as exploratory representations of dietary behavior.

The snack pattern explained 10.02% of the variance and was characterized by milk and milk products, sweetened beverages, bakery items, desserts, pickled fruits, and snacks. The Thai dish pattern explained 9.90% and was loaded on rice, spicy curry, meat, entrée-over-rice dishes, and vegetables. The high-energy pattern explained 9.30% of the variance and loaded primarily on sticky rice and fried meat (Table 3). The pattern labels describe empirical food-group clustering; they do not imply that every food in a pattern has the same nutritional quality. In particular, milk and dairy products are loaded with the snack pattern because they were commonly co-consumed with snack-related foods or used as between-meal items, not because they are considered nutritionally poor.

### 3.3. Associations Between Sociodemographic Factors and Dietary Patterns

#### 3.3.1. Students with Underweight Status

Among the 82 students with underweight status, the snack, Thai dish, and high-energy-pattern models explained 38.3%, 49.0%, and 30.3% of the variance, respectively. Supper intake >3 times per week was positively associated with the snack pattern score (β = 1.04, 95% CI 0.25–1.83, *p* = 0.011).

For the Thai dish pattern, higher year of study (β = 0.33, 95% CI 0.15–0.50, *p* < 0.001), breakfast intake > 3 times per week (β = 0.73, 95% CI 0.33–1.13, *p* < 0.001), and supper intake >3 times per week (β = 0.78, 95% CI 0.21–1.36, *p* = 0.008) were positively associated with the pattern score. Alcohol drinking (β = −1.04, 95% CI −1.87 to −0.21, *p* = 0.014) and sedentary behavior (β = −0.80, 95% CI −1.47 to −0.14, *p* = 0.018) were negatively associated with the Thai dish pattern.

For the high-energy pattern, Islam (versus Buddhism) (β = −0.68, 95% CI −1.21 to −0.14, *p* = 0.013) and GPA >3.00 (β = −0.89, 95% CI −1.63 to −0.15, *p* = 0.018) were negatively associated with the pattern score, whereas sedentary behavior was positively associated (β = 0.92, 95% CI 0.08–1.77, *p* = 0.032) (Table 4).

#### 3.3.2. Students with Excess Body Weight

Among the 98 students with EBW, the snack, Thai dish, and high-energy pattern models explained 40.5%, 43.7%, and 33.6% of variance, respectively.

Female sex (versus male) (β = 0.81, 95% CI 0.07–1.55, *p* = 0.032), reported exercise (β = 0.78, 95% CI 0.34–1.22, *p* = 0.001), and sedentary behavior (β = 1.34, 95% CI 0.76–1.92, *p* < 0.001) were positively associated with snack pattern scores. Higher year of study (β = −0.22, 95% CI −0.41 to −0.04, *p* = 0.017), tea or coffee drinking (β = −0.42, 95% CI −0.84 to −0.01, *p* = 0.048), and alcohol drinking (β = −1.10, 95% CI −1.77 to −0.43, *p* = 0.002) were negatively associated with snack pattern scores.

For the Thai dish pattern, income >5000 THB (β = 0.70, 95% CI 0.20–1.19, *p* = 0.006) and dinner intake >3 times per week (β = 1.02, 95% CI 0.32–1.71, *p* = 0.005) were positively associated with the score. Female sex (β = −1.37, 95% CI −2.16 to −0.58, *p* = 0.001) and supper intake > 3 times per week (β = −1.07, 95% CI −2.06 to −0.08, *p* = 0.034) were negatively associated with the Thai dish score.

GPA >3.00 was negatively associated with the high-energy-pattern score (β = −0.61, 95% CI −1.05 to −0.17, *p* = 0.007) (Table 5).

β, unstandardized regression coefficient; SE, standard error; CI, confidence interval; GPA, grade point average. * *p* < 0.05, ** *p* < 0.01, *** *p* < 0.001. R^^2^^: snack = 0.405; Thai dish = 0.437; high-energy = 0.336. The snack pattern included milk and milk products, sweetened beverages, bakery items, desserts, pickled fruits, and snacks; the Thai dish pattern included rice, spicy curry, meat, entrée-over-rice dishes, and vegetables; and the high-energy pattern included sticky rice and fried meat. Coding: sex (male = 0, female = 1); year of study (first = 1, second = 2, third = 3, fourth = 4); GPA (≤3.00 = 0, >3.00 = 1); income (≤5000 THB = 0, >5000 THB = 1); tea or coffee drinking (no = 0, yes = 1); alcohol drinking (no = 0, yes = 1); exercise (no = 0, yes = 1); sedentary behavior (no = 0, yes = 1); dinner and supper intake (≤3 times/week = 0, >3 times/week = 1). For binary variables, β represents the mean difference in factor score relative to the reference category; for year of study, β represents the mean change in factor score for each one-year increase. The constant is the expected pattern score when all predictors are at their reference values (or year of study = 0).

## 4. Discussion

This study found the population-level coexistence of underweight (22.0%) and EBW (26.3%) among nursing students in Southern Thailand. The observed prevalence should not be interpreted as individual-level DBM; rather, it indicates that both BMI-defined forms of malnutrition are present in the same student population. The prevalence of underweight was higher than the 6.9% reported among healthcare students in Mexico [6], the 7.7% reported in a UK university sample [8], and the 15.2% reported among Brazilian nutrition students [7]. The EBW prevalence was also higher than the combined prevalence of overweight (10.2%) and obesity (5.2%) reported among university students in Macao [18]. Direct comparisons should nevertheless be approached with caution because samples, age ranges, BMI criteria, and dietary measures differ. For example, a study of 125 Brazilian nutrition students identified four dietary patterns explaining 59.8% of dietary variance [7], whereas the three patterns in the present study explained 29.22%, underscoring the exploratory nature of our PCA solution.

Nutritional status distributions differed by year of study. Underweight was descriptively most common among first-year students, whereas EBW was most common among second- and fourth-year students. These cross-sectional differences may reflect variations in adjustment, academic schedules, clinical training, or other unmeasured factors across years of study [20,27]. They do not demonstrate weight change over time and should not be interpreted as a direct Thai equivalent of the U.S. “freshman 15” phenomenon. Longitudinal data are required to examine weight trajectories after university entry [28].

Income was associated with nutritional status: EBW was more frequent among students reporting a monthly income >5000 THB, whereas underweight was more frequent in the lower-income category. This pattern is compatible with, but does not prove, a nutrition-transition explanation in which economic and food environment changes contribute to the coexistence of undernutrition and excess weight in low- and middle-income settings [29,30]. Because income was self-reported, dichotomized, and measured at a single time point, this finding should be interpreted cautiously and not as evidence that income causes either weight status.

The three dietary patterns were distinguishable by their component food groups. The snack pattern included dairy products, sweetened beverages, bakery items, desserts, pickled fruits, and packaged snacks—foods that may be consumed between meals or as quick meal replacements. The high-energy pattern was narrower and was driven primarily by sticky rice and fried meat, which are commonly consumed as meal components. Thus, both patterns may contain energy-dense elements, but they represent different combinations of foods and likely different eating occasions. The Thai dish pattern represented an empirical clustering of rice, spicy curry, meat, entree-over-rice dishes, and vegetables; it should not be interpreted as uniformly healthy because nutritional quality depends on preparation methods, portion sizes, and the specific dishes consumed. The loading of milk and dairy products in the snack pattern describes co-consumption in this sample and does not imply that milk or dairy products are intrinsically nutrient-poor.

Among underweight students, higher Thai dish scores were associated with year of study, breakfast intake, and supper intake, whereas alcohol drinking and sedentary behavior were associated with lower Thai dish scores. High-energy scores were lower among students identifying as Muslim and among those with a GPA >3.00, and higher among those reporting sedentary behavior. Religion was not accompanied by data on dietary restrictions, cultural practices, or food access [31]; therefore, it should be interpreted only as an observed social context correlate, not as evidence for a specific religious mechanism. Similarly, the small number of students reporting alcohol use warrants cautious interpretation of the alcohol-related estimate [32]. The first year of nursing students was a transformative experience, marked by a new sense of freedom, independence, and interpersonal relationships, which can affect dietary behavior change [33].

Among students with EBW, snack pattern scores were higher among female students and among those reporting exercise or sedentary behavior, while higher year of study, tea or coffee drinking, and alcohol drinking were associated with lower snack pattern scores. Thai dish scores were higher among students with higher monthly income and more frequent dinner intake, and lower among female students and those with more frequent supper intake. A GPA > 3.00 was associated with lower high-energy scores. These associations are cross-sectional and may reflect reverse causality or unmeasured factors; for example, students with EBW may have altered their exercise or food choices in response to weight concerns [34,35]. Academic stress, body image, food availability, and university food environments were not measured and are possible explanations that should be tested directly in future work.

The findings point to the potential value of multilevel nutrition promotion. At the individual level, support may address regular meals, healthier snack choices, practical food planning during clinical training, and a reduction in prolonged sedentary time. At the institutional level, campuses may improve the affordability and visibility of balanced meals, reduce the prominence of sugar-sweetened beverages and energy-dense snacks, and provide environments that facilitate physical activity. Such actions should be developed with students and evaluated prospectively rather than inferred solely from the associations observed here.

Because the analysis used separate models for underweight and EBW groups, the study highlights that correlates of dietary pattern scores may differ across these subgroups. However, dietary patterns were not tested as causal determinants of BMI status. Future studies should use longitudinal, multicenter designs and should examine food insecurity, stress, body image, sleep, eating disorder symptoms, campus food availability, and change in weight over time.

### 4.1. Implications for Nursing Practice and Education

Nursing schools can incorporate routine nutrition screening and confidential counseling for students with underweight or EBW status, while avoiding stigmatizing weight-focused approaches. Student wellness programs may include meal planning skills, healthier snack options during clinical placements, support for regular meal timing, and strategies to reduce prolonged sedentary behavior. Campus-level actions could improve access to affordable, balanced meals and drinking water, set nutrition standards for food outlets and vending machines, and support opportunities for physical activity. These interventions should be co-designed with students and assessed for feasibility, equity, and effectiveness.

### 4.2. Strengths and Limitations

This study included students from two randomly selected nursing colleges in Southern Thailand, used standardized anthropometric measurements, and examined dietary patterns rather than single foods. Nevertheless, several limitations require consideration. First, the cross-sectional design precludes temporal or causal inference. Second, the final sample represented two colleges and was predominantly female; findings should not be generalized to all Thai nursing students or other university populations. Third, 24 of 396 returned questionnaires (6.1%) were excluded because of incomplete FFQ data. Dietary-pattern scores could not be calculated for these records, and no comparison between included and excluded records was possible with the available analytical dataset; selection bias is therefore possible. Fourth, intake frequency was self-reported. Random reporting error may attenuate associations, whereas differential under-reporting of energy-dense foods or over-reporting of socially desirable foods by BMI group could either obscure or exaggerate the identified patterns and their associations. Finally, psychological stress, body image, sleep, food insecurity, eating disorder symptoms, and campus food environments were not measured. Underweight should therefore not be interpreted as evidence of an eating disorder.

## 5. Conclusions

BMI-defined underweight and EBW coexisted at the population level among nursing students in Southern Thailand. Three exploratory dietary patterns—snack, Thai dish, and high-energy—showed different associations with sociodemographic and health behavior factors within the underweight and EBW subgroups. The findings do not establish causal effects of dietary patterns on nutritional status; nevertheless, they support tailored student health initiatives, nutrition screening, and healthier university food environments. Longitudinal and multicenter research is needed to confirm these associations and identify modifiable mechanisms.

## Figures and Tables

**Table 1:** Food groups and food items used to derive dietary patterns among nursing students in Southern Thailand, 2023 (n = 372).

Food Groups	Food Items in Each Group
Rice	Plain rice, rice porridge, boiled rice.
Entrée over rice	Rice topped with 2–3 Thai dishes such as coconut milk curry, sour soup, stir-fried vegetables, fried egg, or omelet.
Spicy curry	Curry dishes are cooked with coconut milk, sour curry, and sliced vegetables, and can include fish, pork, or chicken.
Sticky rice	Sticky rice served with deep-fried or grilled chicken or pork.
Thai fast dishes	Fried rice or noodles with chicken or pork and vegetables; rice topped with stir-fried vegetables and meat.
Noodles	Noodles with meatballs, sliced vegetables, and soup.
Thai noodles	Thai rice noodles in fish curry sauce.
Instant noodles	Instant noodles.
Spicy salad	Papaya or mixed fruits with garlic, fish sauce, and chili.
Bakery	White bread, toast with jam, sandwiches, cakes, donuts, and brownies.
Snacks	Potato or banana chips with barbecue flavor, and wafers.
Dessert	Ice cream and Thai desserts, such as fruit or flour-based desserts with sugar and coconut milk.
Sweetened drinks	Iced tea, green tea, and iced flavored fruit drinks.
Meat	Chicken, pork, fish, and shrimp.
Processed meat	Thai-style water fish, meatballs, sausages, and bacon.
Eggs	Boiled eggs, fried eggs, and omelets.
Milk and milk products	Sweetened milk, whole milk, fermented milk, and yogurt.
Fried meat	Deep-fried chicken, pork, fish, and processed meat.
Vegetables	Cucumbers, lentils, kale, cabbage, gourd, Thai morning glory, and green onions.
Pickled fruits	Mango, guava, pineapple, and apple are served with mixed chili–salt–sugar powder

**Table 2:** Factor loadings of food groups in three dietary patterns among nursing students in Southern Thailand, 2023 (n = 372).

Food Group	Snack	Thai Dish	High-Energy
Sticky rice	0.062	0.051	**0.949**
Fried meat	0.159	0.118	**0.885**
Thai noodles	0.221	0.064	0.230
Instant noodles	0.111	−0.084	0.202
Rice	0.046	**0.797**	0.108
Spicy curry	0.124	**0.752**	0.035
Meat	−0.047	**0.600**	0.075
Entrée-over-rice dishes	0.012	**0.405**	0.047
Vegetables	−0.019	**0.314**	0.085
Processed meat	0.128	0.182	0.017
Milk and milk products	**0.729**	0.041	0.022
Sweetened beverages	**0.651**	−0.070	0.127
Bakery items	**0.564**	0.194	−0.056
Desserts	**0.541**	0.030	0.110
Pickled fruits	**0.367**	−0.089	0.026
Snacks	**0.313**	0.101	0.014
Noodles	0.174	−0.129	0.068
Thai fast dishes	0.159	0.098	0.128
Eggs	0.151	−0.012	0.009
Spicy salad	−0.026	0.061	−0.047
Explained variance (%)	10.021	9.895	9.304
Cumulative variance (%)	10.021	19.916	29.220
Eigenvalue	2.004	1.979	1.861

Note: The bold values for the factor loadings in the table are intentionally used to facilitate the identification and grouping of food items within each dietary pattern.

**Table 3:** Sociodemographic characteristics and health behaviors by BMI-defined nutritional status among nursing students in Southern Thailand, 2023 (n = 372).

Variables	Total n (%)	Underweight n (%)	Normal Weight n (%)	EBW n (%)	χ^2^	*p*-Value
Sex					0.276	0.871
Male	27 (7.3)	6 (22.2)	15 (55.6)	6 (22.2)		
Female	345 (92.7)	76 (22.0)	177 (51.3)	92 (26.7)		
Religion					1.414	0.493
Buddhism	286 (76.9)	60 (21.0)	147 (51.4)	79 (27.6)		
Islam	86 (23.1)	22 (25.6)	45 (52.3)	19 (22.1)		
Year of Study					16.128	0.013 *
1st Year	99 (26.6)	28 (28.3)	54 (54.5)	17 (17.2)		
2nd Year	99 (26.6)	18 (18.2)	47 (47.5)	34 (34.3)		
3rd Year	75 (20.2)	15 (20.0)	47 (62.7)	13 (17.3)		
4th Year	99 (26.6)	21 (21.2)	44 (44.4)	34 (34.3)		
GPA					2.303	0.316
≤3.00	73 (19.6)	13 (17.8)	36 (49.3)	24 (32.9)		
>3.00	299 (80.4)	69 (23.1)	156 (52.2)	74 (24.7)		
Income (per month)					11.202	0.004 *
≤5000 THB	299 (80.4)	76 (25.4)	151 (50.5)	72 (24.1)		
>5000 THB	73 (19.6)	6 (8.2)	41 (56.2)	26 (35.6)		
Tea or coffee-drinking					0.990	0.610
Yes	267 (71.8)	62 (23.2)	134 (50.2)	71 (26.6)		
No	105 (28.2)	20 (19.0)	58 (55.2)	27 (25.7)		
Alcohol drinking					6.584	0.034 *^†^
Yes	20 (5.4)	6 (30.0)	5 (25.0)	9 (45.0)		
No	352 (94.6)	76 (21.6)	187 (53.1)	89 (25.3)		
Sedentary behavior					0.573	0.751
Yes	325 (87.4)	70 (21.5)	170 (52.3)	85 (26.2)		
No	47 (12.6)	12 (25.5)	22 (46.8)	13 (27.7)		
Exercise					7.529	0.023 *
Yes	195 (52.4)	40 (20.5)	92 (47.2)	63 (32.3)		
No	177 (47.6)	42 (23.7)	100 (56.5)	35 (19.8)		
Breakfast intake					10.080	0.006 *
≤3 times/week	204 (54.8)	41 (20.1)	120 (58.8)	43 (21.1)		
>3 times/week	168 (45.2)	41 (24.4)	72 (42.9)	55 (32.7)		
Brunch intake					6.602	0.037 *
≤3 times/week	35 (9.4)	12 (34.3)	11 (31.4)	12 (34.3)		
>3 times/week	337 (90.6)	70 (20.8)	181 (53.7)	86 (25.5)		
Lunch intake					2.208	0.332
≤3 times/week	32 (8.6)	10 (31.3)	13 (40.6)	9 (28.1)		
>3 times/week	340 (91.4)	72 (21.2)	179 (52.6)	89 (26.2)		
Dinner intake					5.982	0.050
≤3 times/week	248 (66.7)	48 (19.4)	126 (50.8)	74 (29.8)		
>3 times/week	124 (33.3)	34 (27.4)	66 (53.2)	24 (19.4)		
Supper intake					6.089	0.048 *
≤3 times/week	334 (89.8)	70 (21.0)	170 (50.9)	94 (28.1)		
>3 times/week	38 (10.2)	12 (31.6)	22 (57.9)	4 (10.5)		

Data are presented as n (%). EBW, excess body weight. χ^2^, Pearson chi-square statistic. ^†^ Fisher’s exact test. * *p* < 0.05. Tests are omnibus comparisons across the three nutritional status groups; no post hoc pairwise tests were performed.

**Table 4:** Multivariable linear regression associations with dietary pattern scores among students with underweight status in Southern Thailand, 2023 (n = 82).

Predictors	Snack Pattern			Thai Dish Pattern			High-Energy Pattern		
	β	SE	95% CI	β	SE	95% CI	β	SE	95% CI
Constant	−0.99	1.58	−4.15, 2.17	−2.10	1.15	−4.40, 0.19	1.73	1.46	−1.18, 4.65
Religion	0.34	0.29	−0.23, 0.92	0.15	0.21	−0.26, 0.57	−0.68 *	0.26	−1.21, −0.14
Year of study	−0.10	0.11	−0.34, 0.12	0.33 ***	0.08	0.15, 0.50	−0.07	0.10	−0.28, 0.14
GPA	0.07	0.40	−0.72, 0.87	0.43	0.29	−0.14, 1.01	−0.89 *	0.37	−1.63, −0.15
Alcohol drinking	0.27	0.57	−0.87, 1.41	−1.04 *	0.41	−1.87, −0.21	−0.19	0.52	−1.24, 0.86
Sedentary behavior	−0.30	0.46	−1.22, 0.61	−0.80 *	0.33	−1.47, −0.14	0.92 *	0.42	0.08, 1.77
Breakfast intake	0.19	0.27	−0.39, 0.71	0.73 ***	0.20	0.33, 1.13	0.47	0.25	−0.03, 0.98
Supper intake	1.04 *	0.39	0.25, 1.83	0.78 **	0.28	0.21, 1.36	−0.11	0.36	−0.84, 0.61

β, unstandardized regression coefficient; SE, standard error; CI, confidence interval; GPA, grade point average. * *p* < 0.05, ** *p* < 0.01, *** *p* < 0.001. R^2^: snack = 0.383; Thai dish = 0.490; high-energy = 0.303. The snack pattern included milk and milk products, sweetened beverages, bakery items, desserts, pickled fruits, and snacks; the Thai dish pattern included rice, spicy curry, meat, entrée-over-rice dishes, and vegetables; and the high-energy pattern included sticky rice and fried meat. Coding: religion (Buddhism = 0, Islam = 1); year of study (first = 1, second = 2, third = 3, fourth = 4); GPA (≤3.00 = 0, >3.00 = 1); alcohol drinking (no = 0, yes = 1); sedentary behavior (no = 0, yes = 1); breakfast and supper intake (≤3 days/week = 0, >3 times/week = 1). For binary variables, β represents the mean difference in factor score relative to the reference category; for year of study, β represents the mean change in factor score for each one-year increase. The constant is the expected pattern score when all predictors are at their reference values (or year of study = 0).

**Table 5:** Multivariable linear regression associations with dietary pattern scores among students with excess body weight in Southern Thailand, 2023 (n = 98).

Predictors	Snack Pattern			Thai Dish Pattern			High-Energy Pattern		
	β	SE	95% CI	β	SE	95% CI	β	SE	95% CI
Constant	−1.08	1.10	−3.27, 1.10	2.48	1.17	0.14, 4.81	−0.19	1.14	−2.47, 2.07
Sex	0.81 *	0.37	0.07, 1.55	−1.37 **	0.39	−2.16, −0.58	0.72	0.38	−0.04, 1.49
Year of study	−0.22 *	0.09	−0.41, −0.04	0.14	0.10	−0.05, 0.34	−0.10	0.09	−0.29, 0.09
GPA	−0.02	0.21	−0.45, 0.39	−0.39	0.22	−0.84, 0.05	−0.61 **	0.22	−1.05, −0.17
Income	−0.12	0.23	−0.59, 0.33	0.70 **	0.25	0.20, 1.19	0.06	0.24	−0.42, 0.54
Tea or coffee drinking	−0.42 *	0.21	−0.84, −0.01	−0.29	0.22	−0.74, 0.15	0.23	0.22	−0.20, 0.67
Alcohol drinking	−1.10 *	0.33	−1.77, −0.43	−0.56	0.35	−1.28, 0.14	0.08	0.35	−0.61, 0.77
Exercise	0.78 **	0.22	0.34, 1.22	−0.26	0.23	−0.73, 0.20	−0.30	0.23	−0.76, 0.15
Sedentary behavior	1.34 ***	0.29	0.76, 1.92	−0.19	0.32	−0.81, 0.43	−0.50	0.30	−1.10, 0.10
Dinner intake	0.24	0.32	−0.40, 0.90	1.02 **	0.34	0.32, 1.71	−0.35	0.34	−1.03, 0.32
Supper intake	0.06	0.46	−0.86, 0.99	−1.07 *	0.49	−2.06, −0.08	0.76	0.48	−0.20, 1.72

β, unstandardized regression coefficient; SE, standard error; CI, confidence interval; GPA, grade point average. * *p* < 0.05, ** *p* < 0.01, *** *p* < 0.001. R^^2^^: snack = 0.405; Thai dish = 0.437; high-energy = 0.336. The snack pattern included milk and milk products, sweetened beverages, bakery items, desserts, pickled fruits, and snacks; the Thai dish pattern included rice, spicy curry, meat, entrée-over-rice dishes, and vegetables; and the high-energy pattern included sticky rice and fried meat. Coding: sex (male = 0, female = 1); year of study (first = 1, second = 2, third = 3, fourth = 4); GPA (≤3.00 = 0, >3.00 = 1); income (≤5000 THB = 0, >5000 THB = 1); tea or coffee drinking (no = 0, yes = 1); alcohol drinking (no = 0, yes = 1); exercise (no = 0, yes = 1); sedentary behavior (no = 0, yes = 1); dinner and supper intake (≤3 times/week = 0, >3 times/week = 1). For binary variables, β represents the mean difference in factor score relative to the reference category; for year of study, β represents the mean change in factor score for each one-year increase. The constant is the expected pattern score when all predictors are at their reference values (or year of study = 0).

## Data Availability

De-identified data supporting the findings of this study are available from the corresponding author upon reasonable request, subject to applicable ethical and institutional requirements.
